# Supplementary material for: NMR spectroscopy-based analysis of gallstones of cancerous and benign gallbladders from different geographical regions of the Indian subcontinent
Source: PLoS One. 2023 Jun 23;18(6):e0286979. doi: 10.1371/journal.pone.0286979 (PMC10289436; doi:10.1371/journal.pone.0286979)
Supplement: S1 File — S1 Fig representing 1H NMR spectra of (A) pure cholesterol stone (B) mixed stone and (C) pigment stone depicting the level of cholesterol in different types of stones. S1 Table representing the mean concentration of cholesterol in different regions and the statistical analysis. (DOCX) [file pone.0286979.s001.docx]

**Supplementary Information:**

**NMR spectroscopy-based analysis of gallstones of cancerous and benign gallbladders from different geographical regions of the Indian subcontinent**

**Short Title: Analysis of Gallstones using NMR spectroscopy.**

Mohd Adnan Siddiqui^1,2^, Navneet Dwivedi^2^, Mohammed Haris Siddiqui^1^, S.V. Rana^3^, Anil Sharma^4^, N.R. Dash^5^, Rebala Pradeep^6^, Ranjit Vijayahari^7^, Anu Behari^8^, V.K. Kapoor and Neeraj Sinha^2^

**S1 Fig 1**

**
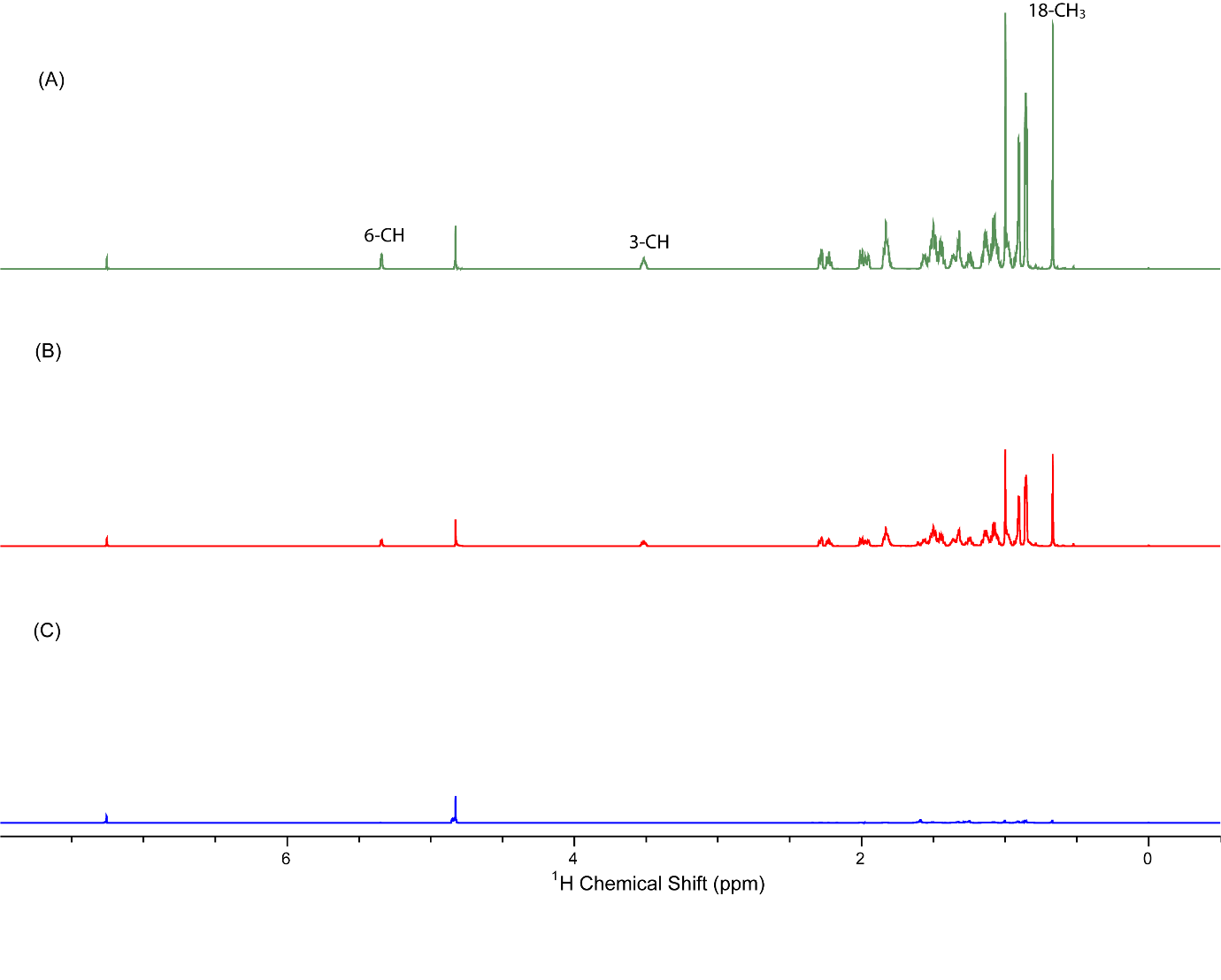
**

**S1 Fig 1.** ^1^H NMR spectra of (A) pure cholesterol stone (B) mixed stone and (C) pigment stone depicting the level of cholesterol in different types of stones.

**S1 Table 1:** Representing the mean concentration of cholesterol in different regions and the statistical analysis.

| **Regions** | **Mean concentration of Cholesterol (mmol/g)** | **p-value** | | | | |
| --- | --- | --- | --- | --- | --- | --- |
|  |  | **Chandigarh** | **Lucknow** | **Kangra** | **West Bengal** | **Delhi** |
| **Chandigarh** | 81.09 | - | NS | NS | NS | NS |
| **Lucknow** | 81.25 | NS | - | NS | NS | NS |
| **Kangra** | 86.16 | NS | NS | - | NS | NS |
| **West Bengal** | 87.71 | NS | NS | NS | - | NS |
| **Delhi** | 82.67 | NS | NS | NS | NS | - |
| **Hyderabad** | 25.2 | **S** | **S** | **S** | **S** | **S** |
| **Thiruvananthapuram** | 17.55 | **S** | **S** | **S** | **S** | **S** |

**# S (significant):** P value <0.05, **# NS (non-significant):** P value >0.05
